# Supplementary material for: Dwarf open reading frame (DWORF) is a direct activator of the sarcoplasmic reticulum calcium pump SERCA
Source: eLife. 2021 Jun 2;10:e65545. doi: 10.7554/eLife.65545 (PMC8203291; doi:10.7554/eLife.65545)
Supplement: Transparent reporting form [file elife-65545-transrepform.pdf]

Westbrook Centre
Milton Road
Cambridge CB4 1YG, UK

P 01223 855340
W elifesciences.org
T @elife

eLife's transparent reporting form

We encourage authors to provide detailed information *within their submission* to facilitate the interpretation and replication of experiments. Authors can upload supporting documentation to indicate the use of appropriate reporting guidelines for health-related research (see EQUATOR Network), life science research (see the BioSharing Information Resource), or the ARRIVE guidelines for reporting work involving animal research. Where applicable, authors should refer to any relevant reporting standards documents in this form.

If you have any questions, please consult our Journal Policies and/or contact us: editorial@elifesciences.org.

Sample-size estimation

- You should state whether an appropriate sample size was computed when the study was being designed
- You should state the statistical method of sample size computation and any required assumptions
- If no explicit power analysis was used, you should describe how you decided what sample (replicate) size (number) to use

Please outline where this information can be found within the submission (e.g., sections or figure legends), or explain why this information doesn't apply to your submission:

For the membrane reconstitution experiments and activity assays, the sample sizes (at least 4 biological replicates) and statistical methods are described in the Materials & Methods section.

To ensure an appropriate study design for the in-cell calcium dynamics, a power analysis (α=0.05) was computed using approximations of anticipated effect size and standard deviation based on previous investigations. This proposal relied primarily on intracellular calcium handling measurements, and using approximations for these parameters the Power analysis suggests that ~15 cells per experimental group are needed to achieve a power of 0.80. Three major experimental protocols were designed to study the effect DWORF and PLN on SERCA function in different conditions (total 40 experiments). Based on our preliminary results, the rate of success of experiments and the amount of data that we obtained during a single experiment, we estimated that we needed to conduct 40 experiments to achieve the aim of this study. On average, ~6 cells yielded reliable data per experiment. Altogether, 240 cells needed to be analysed for this study (40 experiments x 8 cells per group as determined from Power analysis).

For the FRET experiments, we did not perform an explicit power analysis, rather, we conducted a standard assay with a practical sample size and tested whether the results were statistically significant.

Replicates

- You should report how often each experiment was performed
- You should include a definition of biological versus technical replication
- The data obtained should be provided and sufficient information should be provided to indicate the number of independent biological and/or technical replicates
- If you encountered any outliers, you should describe how these were handled
- Criteria for exclusion/inclusion of data should be clearly stated

eLife Sciences Publications, Ltd is a limited liability non-profit non-stock corporation incorporated in the State of Delaware, USA, with company number 5030732, and is registered in the UK with company number FC030576 and branch number BR015634 at the address Westbrook Centre, Milton Road, Cambridge CB4 1YG.

- High-throughput sequence data should be uploaded before submission, with a private link for reviewers provided (these are available from both GEO and ArrayExpress)

Please outline where this information can be found within the submission (e.g., sections or figure legends), or explain why this information doesn't apply to your submission:

For the membrane reconstitution experiments, the sample sizes and statistical methods described for each experiment refer to the number of reconstitutions and activity assays (biological replicates).

For the in-cell calcium dynamics, one-to-two experiments were conducted every week during a five-month period (total 40 experiments). Biological replicates are measurements of biologically distinct samples such as Control, DWORF and PLN. Technical replicates are repeated measurements of the same sample e.g. DWORF. In the three major experimental protocols, 8 independent biological replicates were conducted. Technical replicates were ranged from 18 to 46 independent measurements.

FRET experiments involved 3 separate transfections on 3 different days, with 2 separately permeabilized technical replicates each. Each replicate resulted in a FRET binding curve composed of fluorescence measurements of approximately 400 cells.

Statistical reporting

- Statistical analysis methods should be described and justified
- Raw data should be presented in figures whenever informative to do so (typically when N per group is less than 10)
- For each experiment, you should identify the statistical tests used, exact values of N, definitions of center, methods of multiple test correction, and dispersion and precision measures (e.g., mean, median, SD, SEM, confidence intervals; and, for the major substantive results, a measure of effect size (e.g., Pearson's r, Cohen's d)
- Report exact p-values wherever possible alongside the summary statistics and 95% confidence intervals. These should be reported for all key questions and not only when the p-value is less than 0.05.

Please outline where this information can be found within the submission (e.g., sections or figure legends), or explain why this information doesn't apply to your submission:

For the ATPase activity assays, comparison of KCa and Vmax values was carried out using one-way analysis of variance (between subjects), followed by the Holm-Sidak test for pairwise comparisons.

For the in-cell calcium dynamics, data are presented as mean ± SEM of n measurements of individual cell. Statistical comparisons between groups were performed with the Student's t test for paired or unpaired data sets. Differences were considered statistically significant at $P<0.05$. Comparisons among multiple sample groups were performed with one-way analysis of variance (ANOVA). Statistical analysis and graphical representation of averaged data were carried out on OriginPro7.5 software (OriginLab, USA). Figure 3C: CTRL 46 cells, DWORF 30 cells and PLN 28 cells. Total 25 experiments. Figure 4B: CTRL 27 cells, DWORF 25 cells and PLN 18 cells. Total 12 experiments. Figure 4D: CTRL 25 cells and DWORF 20 cells. Total 6 experiments.

For FRET experiments, statistical significance was determined by an unpaired Student's T-test.

eLife Sciences Publications, Ltd is a limited liability non-profit non-stock corporation incorporated in the State of Delaware, USA, with company number 5030732, and is registered in the UK with company number FC030576 and branch number BR015634 at the address Westbrook Centre, Milton Road, Cambridge CB4 1YG.

(For large datasets, or papers with a very large number of statistical tests, you may upload a single table file with tests, Ns, etc., with reference to sections in the manuscript.)

Group allocation

- Indicate how samples were allocated into experimental groups (in the case of clinical studies, please specify allocation to treatment method); if randomization was used, please also state if restricted randomization was applied
- Indicate if masking was used during group allocation, data collection and/or data analysis

Please outline where this information can be found within the submission (e.g., sections or figure legends), or explain why this information doesn't apply to your submission:

For the membrane reconstitution experiments and activity assays, samples were grouped according to the absence or presence of the DWORF peptide.

For the in-cell calcium dynamics and FRET experiments, samples were allocated into experimental groups based on expression of specific regulators of SERCA function: DWORF and PLN.

Additional data files ("source data")

- We encourage you to upload relevant additional data files, such as numerical data that are represented as a graph in a figure, or as a summary table
- Where provided, these should be in the most useful format, and they can be uploaded as "Source data" files linked to a main figure or table
- Include model definition files including the full list of parameters used
- Include code used for data analysis (e.g., R, MatLab)
- Avoid stating that data files are "available upon request"

Please indicate the figures or tables for which source data files have been provided:

Data are available upon request.

eLife Sciences Publications, Ltd is a limited liability non-profit non-stock corporation incorporated in the State of Delaware, USA, with company number 5030732, and is registered in the UK with company number FC030576 and branch number BR015634 at the address Westbrook Centre, Milton Road, Cambridge CB4 1YG.
